# HIV mortality in urban slums of Nairobi, Kenya 2003–2010: a period effect analysis

**DOI:** 10.1186/1471-2458-13-588

**Published:** 2013-06-17

**Authors:** Samuel Oji Oti, Michael Mutua, George S Mgomella, Thaddaeus Egondi, Alex Ezeh, Catherine Kyobutungi

**Affiliations:** 1 African Population and Health Research Center, P.O. Box 10787–00100, GPO Nairobi, Kenya; 2 Department of Global Health, Amsterdam Institute for Global Health and Development, Academic Medical Center, University of Amsterdam, P.O. Box 22700, 1100, DE, Amsterdam, The Netherlands; 3 Department of Public Health and Primary Care, University of Cambridge, Worts’ Causeway, Cambridge CB1 8RN, UK; 4 Department of Public Health and Clinical Medicine, Epidemiology and Global Health, Umea University, SE-901 85, Umea, Sweden

**Keywords:** Verbal autopsy, Antiretroviral therapy, HIV and AIDS-related death, Demographic surveillance system, Nairobi, Sub Saharan Africa

## Abstract

**Background:**

It has been almost a decade since HIV was declared a national disaster in Kenya. Antiretroviral therapy (ART) provision has been a mainstay of HIV treatment efforts globally. In Kenya, the government started ART provision in 2003 with significantly scale-up after 2006. This study aims to demonstrate changes in population-level HIV mortality in two high HIV prevalence slums in Nairobi with respect to the initiation and subsequent scale-up of the national ART program.

**Methods:**

We used data from 2070 deaths of people aged 15–54 years that occurred between 2003 and 2010 in a population of about 72,000 individuals living in two slums covered by the Nairobi Urban Health and Demographic Surveillance System. Only deaths for which verbal autopsy was conducted were included in the study. We divided the analysis into two time periods: the “early” period (2003–2006) which coincides with the initiation of ART program in Kenya, and the “late” period (2007–2010) which coincides with the scale up of the program nationally. We calculated the mortality rate per 1000 person years by gender and age for both periods. Poisson regression was used to predict the risk of HIV mortality in the two periods while controlling for age and gender.

**Results:**

Overall, HIV mortality declined significantly from 2.5 per 1,000 person years in the early period to 1.7 per 1,000 person years in the late period. The risk of dying from HIV was 53 percent less in the late period compared to the period before, controlling for age and gender. Women experienced a decline in HIV mortality between the two periods that was more than double that of men. At the same time, the risk of non-HIV mortality did not change significantly between the two time periods.

**Conclusions:**

Population-level HIV mortality in Nairobi’s slums was significantly lower in the approximate period coinciding with the scale-up of ART provision in Kenya. However, further studies that incorporate ART coverage data in mortality estimates are needed. Such information will enhance our understanding of the full impact of ART scale-up in reducing adult mortality among marginalized slum populations in Kenya.

## Background

The global HIV epidemic is one of the worst to affect humanity over the last century [1]. Sub-Saharan Africa (SSA) has borne the brunt of this epidemic where HIV/AIDS has been the leading cause of death in the last decade −1.8 million deaths occurred in the subcontinent from HIV/AIDS in 2010 alone [2]. Kenya is one of the countries in SSA that has suffered significantly from the burden of HIV. There are about 1.4 million adults living with HIV in Kenya and the national prevalence was 6.4 percent among adults aged 15 – 49 years in 2008 [3,4]. This is down from the estimated prevalence of between 8 and 10 percent in the late 1990s [5,6] and a prevalence of about 7 percent in 2002 [7]. Despite this decline, about 80,000 new HIV infections and 110,000 HIV related deaths are believed to occur in Kenya yearly [4,6]. Women aged 15 – 49 years and urban residents in Kenya appear to be disproportionately burdened with HIV compared to men and rural residents [3].

Among urban residents, there is limited knowledge on the impact of HIV in slum settlements in Kenya and indeed other countries in SSA, although it is known that urban averages hide gross intra-urban health inequalities [8]. Slums are of increasing interest because they are typically home to majority of urban dwellers in low- and middle income countries. In Kenya, it is estimated that between 60 to 70 percent of the urban population resides in slums [9]. These slums are typically underserved by social amenities such as water and sanitation services and have limited access to quality preventive and curative health services. Additionally, slums are characterised by high levels of poverty, unemployment, and insecurity.

Unfortunately, not much is known about the impact of HIV mortality among the urban poor who reside in vast slums across most urban centres in Kenya [10]. It would be anticipated that slum residents are more adversely affected by the HIV epidemic than non-slum residents since they generally fare worse off on other health indicators [8,11]. For example, a sero-prevalence study conducted in 2008 in two slums in Nairobi among a sample of 2,000 adults aged 18 years and above, found a prevalence of 11.5 percent [12]. This figure is almost double the national average and 1.5 times the prevalence for the entire Nairobi city [3]. In spite of their disadvantaged situation, slum residents in Kenya may have benefited from local and national HIV prevention and control initiatives. Kenya is one of 22 countries, out of the 33 globally, where HIV incidence has declined [2]. This success can be largely attributed to several factors: the global response (HIV prevention, care, treatment and impact mitigation) to the epidemic including the launch of UNAIDS in 1996, the targeting of HIV/AIDS as one of the Millennium Development Goals in 2000; the launch of the Global Fund to fight AIDS, TB and Malaria in 2002; and the announcement of U.S President’s Emergency Plan for AIDS Relief (PEPFAR) by President Bush in 2003, among others [13-15]. Locally, the launch of the National AIDS and STI Control Programme (NASCOP) and the National AIDS Control Council (NACC) in 1987 and 1999 respectively were crucial steps for guiding policy direction and mobilizing resources for the fight against HIV/AIDS in Kenya [16,17].

Overall however, many resource poor countries like Kenya rely heavily on foreign aid in their fight against HIV/AIDS [18]. According to a UNAIDS report, this high dependence on foreign aid suggests a challenge in national ownership and sustainability of the HIV response in these countries [19]. There is therefore growing demand by the international community for greater country ownership of the burden of care and support of those infected and affected by HIV/AIDS in such settings. This is more so in view of the ongoing global economic downturn as well as emergence of competing health priorities such as the rising burden of non-communicable diseases [20]. Empirical evidence on the impact of the HIV response may contribute, among other factors, to increased country ownership among national governments.

One way to demonstrate the impact of the HIV response is through population-level data on mortality attributable to HIV/AIDS. Unfortunately, most resource-poor countries that bear the highest burden of HIV also lack complete vital registration-type data needed to generate actual population-level figures such as national HIV mortality estimates [21-23]. As a result, much of what is known about population-level mortality in such settings, are based on statistical modelling and estimation [24-26]. In the absence of vital registration systems and data on causes of death, Health and Demographic Surveillance Systems (HDSS) provide a useful platform for contributing to the understanding of the population-level mortality. Typically, HDSS monitor and track demographic and health indicators in a population within a defined geographic area [27]. Usually, HDSS collect data on vital events including births, death and migration within the population under surveillance and at regular intervals [27]. This paper is based on data from the Nairobi Urban Health and Demographic Surveillance System (NUHDSS) in Kenya. This HDSS covers two slums located in the heart of Nairobi. We analysed data on mortality and causes of death in the NUHDSS that was collected between 2003 and 2010.

The period under study, 2003 – 2010, is of particular significance because it traverses two important milestones. First, the initiation of the national antiretroviral therapy (ART) program in Kenya occurred just before 2003 and by then, only 5 percent of those needing ART were receiving treatment [7,28]. Second, in 2006, the Government of Kenya announced that antiretroviral drugs would be provided for free in all public hospitals and health centres nationwide [29]. Since then, the national coverage of ART has increased significantly. Public-sector sites providing ART proliferated from 30 in 2004 to over 1000 in 2011 [30,31]. By 2009, the national coverage of ART was estimated to have reached 70 percent of those needing ART [4]. However, the World Health Organization (WHO) changed its treatment guidelines and recommended earlier start to treatment. Thus, it is estimated that by 2010, only half of all Kenyans in need of ART were receiving it, going by the government figures and the new WHO treatment guidelines [32]. Overall therefore, the first four years of our study period (2003–2006) could be said to coincide with the early period of the national ART program while the last four years (2007–2010) coincides with the scaling up of the program. These timelines are important for our analysis because ART scale-up has been demonstrated in a few studies in SSA to have an impact on population-level mortality [33-35]. We thus expect that the scaling up of the national ART program in Kenya could potentially have an influence on population-level mortality in our study areas.

Ideally, we would have preferred to match the analysis periods with specific ART coverage milestones and data from our study area. However, such data is unavailable mostly because the informal nature of the slums implies that there are no centrally located public health facilities serving these communities from which such data could be obtained. The private-for-profit and non-profit health facilities serving these settlements have weak health management information systems and no coordinating mechanisms to aggregate the data generated. In addition, some patients are known to seek care far away from their areas of residence to avoid stigma and hence facility-based ART coverage data may not accurately reflect population coverage. Nonetheless, we anticipate that since our study areas are located in the heart of the capital city of Kenya, then the study communities could very well have benefitted from the national ART program. This study aims to demonstrate changes in population-level HIV mortality in two high HIV prevalence slums in Nairobi with respect to the approximate timings of the initiation and subsequent scale-up of the national ART program.

## Methods

### Study area and population

We use cause of death data from 2070 deaths that occurred in the NUHDSS between 2003 and 2010 among males aged between 15 and 54 years, and females aged between 15 and 49 years, the age groups most affected by HIV/AIDS. Since 2002, the African Population and Health Research Center (APHRC), a regional research institution head-quartered in Nairobi-Kenya, has been operating the NUHDSS. The NUHDSS covers the two slums of Viwandani and Korogocho both located less than 10 kilometres from the Nairobi City centre [36]. Viwandani and Korogocho occupy an area of 0.45 and 0.52 km^2^ respectively. In total, the population under surveillance in both slums is about 72,000 people, living in 28,500 households, and originating from over 15 ethnic groups [36]. The population in Korogocho is mainly comprised of long-term settlers engaged in informal employment such as domestic work, petty trading and casual work. Viwandani is mainly comprised of semi-skilled labour migrants working in the neighbouring industrial area.

### Cause of death data in the NUHDSS

As part of the activities of the NUHDSS, data on individual and household core demographic events including birth, death, in-migration, and out-migration are collected at four-monthly intervals known as data collection rounds. These data are collected by trained field interviewers drawn from the study area and who have a minimum of a high school certificate or 12 years of formal schooling. These field interviewers identify and interview key informants, usually the head of the household or other adult member of the household, who provide information about the status of all known household members listed in the last visit and any visitors to the household since the last visit. As many as three repeat visits are made by the field interviewers to locate a ‘credible respondent’ after which the status of the household members is updated as “unknown” for that round, if an interview cannot be done.

Additionally, the NUHDSS integrates the Verbal Autopsy (VA) process for ascertaining cause of death. VA is the process of retrospectively interviewing the primary caregivers of recently deceased persons in order to gain an understanding about the circumstances surrounding and leading up to death [37]. VA can be used to provide information on causes of death in resource-constrained settings where vital registration systems are often incomplete or non-existent. Details of this process in the NUHDSS have been published elsewhere [37,38]. In brief, trained interviewers visit a household, on average within three months after a death has occurred, to conduct a verbal autopsy interview with a credible respondent –usually a spouse or other close family member who would be aware of the circumstances surrounding death. The interview is conducted using a semi-structured data collection tool that mostly inquires about probable symptoms and signs that the deceased may have portrayed before death. The completed tools are then passed to a pair of physicians who independently review each case and assign a probable cause of death based on WHO International Statistical Classification of Diseases and Related Health Problems (ICD-10) [39]. The causes of death assigned by each physician are then compared and those cases that are in agreement are recorded as the final causes of death. If there are any ‘disagreements’ the physicians meet and hold a consensus meeting arbitrated by a third physician. Where there is no agreement even after this meeting, such cases are coded at “indeterminate”. The cause of death data used for this paper was retrospectively reviewed by two pairs of physicians over a period of about one month from August to September 2011. Prior to this, each physician received three-days of training in ICD-10 coding.

### Period effect analysis

All analyses were performed using STATA Statistical Software Release 11 (StataCorp LP, College Station, Texas, US). HIV- and all-cause mortality was calculated by aggregating deaths and person-years of observation for the two periods: 2003–2006 (early) and 2007–2010 (late). Residents contributed person time as long as they were living in the NUHDSS area. Residents stopped contributing time if they out-migrated and resumed contributing if they re-entered the NUHDSS area. Mortality rates by gender and age group were computed and compared for the early and late periods. Additionally, a Poisson regression model was used to estimate and compare the incidence (mortality) risk ratio or IRR (for all-cause and HIV mortality) between the two periods, while controlling for age and gender. In the regression model, we modelled the counts of deaths as a function of select individual-level characteristics –age, gender and period using the offset of person years.

In order to determine if any potential changes in mortality between the two observation periods could have been attributable to an underlying change in the population structure, we standardized mortality rates between the two observation periods using the un-weighted average of the two population compositions as the standard. To do this, first the percent distribution for each period, and then the average percent by gender and age categories were computed. Decomposition of the difference between the two period rates into a part due to compositional differences and a part due to differences in rates was done, using the *dstdize* command in STATA which uses a weighted average of the two populations as the standard. A Poisson regression-based standardization for count data was also necessary to control for differences in compositional variables such as gender and age. This analysis yielded the changes in all-cause, HIV and non-HIV mortality between the two periods, the differences of which would not be explained by changes in underlying population structure between the two periods.

### Missing or indeterminate causes of death

Overall there were 2070 deaths of which 560 (27%) deaths were classified as indeterminate or missing. In the previously described analyses, indeterminate or missing causes of death were classified as ‘non-HIV’. However, an imputation model was applied to distribute missing or indeterminate cause of death data to various causes of death. We derived an imputation model for cause-of-death that included the variables of sex, age, slum and year of death. The variable year was included in the model to capture any trends in mortality for different causes of death. This imputation model was implemented using STATA’s *ice* program which uses Multiple Imputations by Chained Equations (MICE) [40]. This approach creates multiple imputations as opposed to single imputation and this helps account for statistical uncertainty in imputation.

### Ethical considerations

We utilised routine data collected every four months as part of the NUHDSS. In order to operate the NUHDSS, APHRC applied for, and received approval from the Kenya Medical Research Institution’s National Ethics Review Committee (KEMRI/NERC). All VA data generated from the NUHDSS is treated as strictly confidential and access is not granted to anyone outside of the research team except in accordance with APHRC’s data sharing policy [41].

## Results

### Mortality rates

#### All cause vs. HIV mortality

All-cause mortality declined from 6.7 deaths per 1000 person years (PY) in the early period to 6.1 in the late period (Table 1). Although women had a higher mortality rate in the early period compared to men (7.5 versus 6.1 deaths per 1000 PY respectively), they showed an overall larger decline in all-cause mortality rate (25 percent reduction or 1.9 deaths per 1000 PY) between the early and late period. For men, all-cause mortality increased slightly by 6.5 percent (or 0.4 deaths per 1000 PY) between the early and late period. All-cause mortality also declined in the late period across all age-groups with the largest decline seen in the 35–44 years age-group.

**Table 1:** **All**-**cause and HIV related mortality rates by period**, **gender and age group**

	**All**-**cause mortality**							**HIV related mortality**						
	**2003** - **2006**			**2007** - **2010**				**2003** - **2006**			**2007** - **2010**			
	**Person years**	**Deaths**	**Mortality Rate**	**Person years**	**Deaths**	**Mortality rate**	**Change in rate**	**Person years**	**Deaths**	**Mortality rate**	**Person years**	**Deaths**	**Mortality rate**	**Change in rate**
Overall	158472.7	1057	6.7	165110.5	1013	6.1	−0.6	158472.7	398	2.5	165110.5	287	1.7	−0.8
Gender														
Female	64458.3	483	7.5	69285.8	386	5.6	−1.9	64458.3	224	3.5	69285.8	138	2.0	−1.5
Male	94014.4	574	6.1	95824.6	627	6.5	0.4	94014.4	174	1.9	95824.6	149	1.6	−0.3
Age group														
15-24	56656.6	174	3.1	54497.3	152	2.8	−0.3	56656.6	43	0.8	54497.3	21	0.4	−0.4
25-34	62350.5	411	6.6	64458.7	380	5.9	−0.7	62350.5	168	2.7	64458.7	102	1.6	−1.1
35-44	27950.4	308	11.0	31882.7	310	9.7	−1.3	27950.4	129	4.6	31882.7	108	3.4	−1.2
45-54	11515.1	164	14.2	14271.8	171	12.0	−2.3	11515.1	58	5.0	14271.8	56	3.9	−1.1

HIV mortality declined significantly from 2.5 deaths per 1000 PY in the early period to 1.7 deaths per 1000 PY in the late period (or a 32 percent reduction). HIV mortality was almost double among females (3.5 deaths per 1000 PY) in the early period compared with men (1.9 per 1000 PY). However, HIV mortality declined by 43 percent between the early and late period among women compared to a decline of 16 percent for men during the same period. This significantly reduced the gender gap in HIV mortality in the late period.

Consistent with the age profile of HIV prevalence in most sub-Saharan African countries, HIV mortality increased with age in both the early and late periods. However, in the late period, HIV mortality was cut by half in the youngest age group (15–24) and by 41 percent in the age-group 25–34 compared to declines of 22–26 percent in the older age groups.

#### Period effect on mortality

Using Poisson regression, the period effect on all-cause, HIV and non-HIV related mortality was assessed, controlling for age and gender. The results are shown in Table 2. Males had an 18 percent lower risk of dying from all-causes compared with females (p < 0.0001). The IRR from all-cause mortality increased linearly with age and this was true for both males and females. In all instances, the differences in IRR by age were statistically significant. There was 14 percent reduction in IRR from all-cause mortality in the late period compared to the early one, controlling for age and gender (p < 0.0001). These declines appeared to have been driven exclusively by reductions in female IRR which decreased by 32 percent in the late period compared to that of males which increased marginally by 2 percent between the early and late periods.

**Table 2:** **Incidence risk ratios for all**-**cause and HIV related mortality rates by time period**

	**All**-**cause deaths**						**HIV**-**related deaths**					
	**Overall**	**p**-**value**	**Female**	**p**-**value**	**Male**	**p**-**value**	**Overall**	**p**-**value**	**Female**	**p**-**value**	**Male**	**p**-**value**
Male (*ref*: *Female*)	0.82	***	-	-	-	-	0.44	***	-	-	-	-
Age (*ref*: *15*–*24*)												
25-34	2.22	***	3.17	***	1.59	***	3.00	***	3.00	***	3.24	***
35-44	3.77	***	5.11	***	2.84	***	5.36	***	4.78	***	6.76	***
45-54	4.83	***	6.14	***	3.76	***	5.65	***	4.06	***	8.16	***
2007-2010 (*ref*: *2003*–*2006*)	0.86	**	0.68	***	1.02	0.784	0.47	***	0.48	***	0.47	***

**p<0.001, ***p<0.0001.

For HIV mortality, IRR was 56 percent lower in males compared with females over the entire period. IRR from HIV mortality among males increased with advancing age, whereas for females, the IRR from HIV mortality was highest in the 35–44 years age group. The risk of dying from HIV was 53 percent lower in the late period compared with the early period, controlling for age and gender. This risk reduction was similar for both males and females at 53 and 52 percent respectively.

#### Period-standardization effect on mortality

Table 3 shows the period-standardized mortality rates and IRR for all-cause, HIV- and non-HIV mortality by observation period. Of particular note is the finding that on one hand, HIV mortality declined in the late period for males, females, and across all age groups. However, on the other hand, non-HIV mortality either increased or remained the same except among females where it declined marginally. Further, the IRR for all-cause mortality was significantly reduced by 12 percent in the late period. Whereas the HIV mortality risk was 35 percent less in the late period (p < 0.0001). Finally, the risk of non-HIV mortality did not change significantly in the late period.

**Table 3:** Period-standardized mortality rates for all-cause, HIV-related and non-HIV mortality

		**All cause mortality**		**HIV related mortality**		**Non-HIV mortality**	
		**2003-2006**	**2007-2010**	**2003-2006**	**2007-2010**	**2003-2006**	**2007-2010**
Overall mortality		6.7	6.1	3.3	2.3	3.4	3.8
Gender	Male	3.6	3.8	1.4	1.0	2.2	2.8
	Female	3.0	2.3	1.9	1.3	1.1	1.0
Age							
	15-24	1.1	0.9	0.4	0.2	0.7	0.7
	25-34	2.6	2.3	1.4	0.8	1.2	1.5
	35-44	2.0	1.9	1.1	0.9	0.9	1.0
	45-54	1.0	1.0	0.5	0.4	0.5	0.6
Regression based standardization							
		IRR	(p-value)	IRR	(p-value)	IRR	(p-value)
Female		1.22	***	2.34	***	0.70	***
25-34		2.19	***	3.85	***	1.56	***
35-44		3.67	***	7.58	***	2.28	***
45-54		4.68	***	8.78	***	3.19	***
2007-2010		0.88	***	0.65	***	1.10	

***p < 0.0001.

## Discussion

We set out to determine if there is any evidence of reduction in population-level HIV mortality among a population of slum dwellers living in areas covered by a health and demographic surveillance system in Nairobi, over a period that coincides with the initiation and mass scale-up of the national ART program in Kenya. Overall, this study found significant declines in HIV mortality in the late period (2007–2010) that coincided with the scaling up of the national ART program in Kenya compared with the early period (2003–2006) just following the commencement of the program. This decline occurred despite the very high prevalence of HIV in the study community, 11.5 percent in 2008 [12] and high mortality that have been previously attributable to HIV [38]. Recent work in South Africa and Malawi have shown decline in mortality at the population level following the scale-up of ART in the study populations [33,34]. Like both of these studies, our study was conducted using mortality data from surveillance of a large population and the timing of the decline in mortality coincides with the scale up of ART.

However, unlike both of these studies, the lack of ART coverage and uptake data specific to our study areas is a major limitation to our study. We also lack data on treatment initiation which may have an impact on mortality with or without ART scale up. Overall, we have had to rely on the timelines of ART scale-up nationally. In spite of this limitation, it is plausible that the decline in population-level mortality in our study area could be attributable to the national ART scale-up program. This is because overall trends in mortality in the surveillance areas show that, apart from HIV-related causes, all other causes of death were either stable or increased over the eight years covered by our analysis. In addition, we are not aware of any other major health interventions in the study area during the period covered by our analysis. More importantly, reduction in HIV mortality in the late period was more than double the reduction in all-cause mortality. Given the significant declines in the late period of the risks of all-cause mortality and HIV mortality, as well the lack of change in the risk of non-HIV-related deaths, we can infer that the observed declines in overall mortality in the late period is due largely from the declines in HIV-related deaths. And we believe that this decline in HIV deaths is most likely due to the scaling up of ART in public health facilities in Kenya just after 2006.

Another interesting finding from our study was that declines in HIV mortality were much larger for women than for men. Specifically, it was 2.7 times larger for females than for males ((1.5/3.5 = 42.86%) / (0.3/1.9 = 15.79%) = 2.7). This was dissimilar to the findings of the study in rural South Africa where approximate declines in HIV mortality of 22 percent and 29 percent was observed in adult women and men respectively. The study in Malawi did not find any gender differences in mortality reduction. In our study, the excess female disadvantage in HIV-related deaths reduced from 84 percent in the early period to only 25 percent in the late period (see Table 1). This observed reduction in excess female mortality in the late period could suggest that gender inequities in HIV-related deaths are being successfully addressed by HIV control programs such as the national ART scale-up program in Kenya. On the other hand, we found that all-cause mortality for men increased slightly in the late period. This might be attributable to the observation that injuries and accidents, which account for the largest proportion of deaths among adult male residents in the slum area, actually increased over the entire study period as the competing risk from HIV declined. In terms of age differentials, we found the largest drop (50 percent) in HIV-related deaths among younger people aged 15–24 years. This is a very important finding in this setting since younger adults are more likely to be unemployed and hence less likely to afford treatment compared to older persons. The finding could mean that the relatively wide scale provision of free ART in the late period was particularly beneficial to this age group.

Aside from the lack of data on ART coverage and uptake, our study has a few other limitations. First, we do not have mortality data prior to 2003 so we were unable to determine the effect of secular trends on our results. However, available estimates for Kenya show that since 1990, AIDS deaths have been on an upward trajectory until after 2005 [42]. Also, we could not conduct a sensitivity analysis for our verbal autopsy data so it is impossible to say that the observed changes were not due to misclassification of HIV deaths. To perform a sensitivity analysis we would need either sero-prevalence data among those in the study or health facility data. However, the only sero-prevalence study in the area was done towards the end our study period in 2008 [12]. We attempted to ‘verify’ deaths attributed to HIV according to VA by analysing data for those individuals who had participated in the sero-prevalence study and who had died within the study period. There were 61 of such cases −25 people or 41 percent of those who died were previously diagnosed as HIV positive. The sensitivity of VA in ‘diagnosing’ HIV deaths among those who were known HIV positive was 64 percent (16 HIV deaths out of 25 HIV positive persons). The specificity of VA in diagnosing non-HIV deaths among those who were HIV negative was 81 percent (29 non-HIV deaths out of 36 HIV negative persons). These findings are consistent with previous studies which have shown that verbal autopsies are considerably valid in detecting HIV deaths especially among adults though underestimation of HIV deaths is not uncommon [43-45]. Generally, the accuracy of verbal autopsies interpreted by physicians is said to have an acceptable level of diagnostic accuracy, at the population level, if sensitivity and specificity are at least 50 percent and 90 percent respectively [46].

One other potential limitation of our study is that the absence of sero-prevalence data for the period under study makes it impossible to determine if the observed findings were due to any differential out-migration of HIV positive persons from the slum areas. The study in South Africa did not find any evidence of differential out-migration between HIV positive persons, HIV negative persons and those whose HIV status was unknown. We do not have any reason to believe that there was any differential migration in our study area during the study period. Further, the overall trends in out-migration rates remained fairly stable in the study area over both the early and late periods (see Table 4). This is with the exception of 2004 when there was forceful demolition by local authorities of slum structures built near railway lines and under main power lines in one of the slums.

**Table 4:** **Open cohort of females aged 15**–**49 years and males aged 15** – **54 years**, **2003**–**2010**, **NUHDSS**

**Females aged 15 - 49**	**2003**	**2004**	**2005**	**2006**	**2007**	**2008**	**2009**	**2010**
Population at start of year	12686	14566	13983	14777	15219	15360	16318	17569
Entered age group during year	313	330	340	293	390	345	365	386
In-migrated (from outside *DSA) during year	4199	4620	4134	3700	3456	4048	4551	4086
Died during year	153	118	106	108	86	88	91	119
Exited age group during year	83	55	68	69	101	86	168	69
Lost to follow-up during year (out-migrations)	3125	5824	3650	3465	3770	3363	3525	3907
Total person time contributed each year	16191.3	16118.1	16663.6	17086.0	17527.0	17702.3	19789.4	19989.1
**Out-migration rates	0.19	0.36	0.22	0.20	0.22	0.19	0.18	0.20
**Males aged 15 - 54**	**2003**	**2004**	**2005**	**2006**	**2007**	**2008**	**2009**	**2010**
Population at start of year	20245	22389	21019	21721	22021	22152	23116	24434
Entered age group during year	324	335	371	301	372	314	331	386
In-migrated (from outside *DSA) during year	4997	5658	4767	4545	4478	5107	5679	5121
Died during year	158	161	125	130	160	136	153	178
Exited age group during year	106	136	71	227	111	173	124	164
Lost to follow-up during year (out-migrations)	4259	7980	4528	4300	4653	4363	4480	4866
Total person time contributed each year	24371.4	23708.8	24169.6	24393.4	24874.4	25288.1	27321.2	27680.4
**Out-migration rates	0.17	0.34	0.19	0.18	0.19	0.17	0.16	0.18

*DSA Demographic Surveillance Area.

**Out-migration rates were calculated by dividing the number of persons who out-migrated each year by the total person years contributed by those present at the start of the year and those who in-migrated during the year.

**Figure 1:**
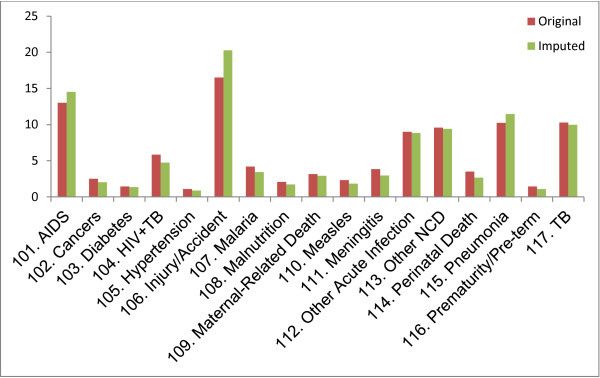
Plot showing imputed versus original causes of death, NUHDSS 2003 – 2010.

Again, about 27 percent of all deaths in this study were not successfully coded or assigned a cause of death, that is, either the physicians were unable to arrive at a consensus on the causes of death, or there was no credible respondent available to conduct a VA interview. To determine the impact of this on our findings, we applied an imputation model to causes of death as described in the methods section of this paper. Overall, the pattern of cause-specific mortality for the imputed model did not differ significantly from that in which the causes of death are known (see Figure 1). We went further to estimate the mortality rates in the early and late periods using the imputed data (see Additional file 1). The trends in HIV mortality in the early and late period using the imputed data were similar to the trends described previously for the original data.

The major strength of our study is the large number of cases considered in the analysis. This is borne out of the advantage provided by a health and demographic surveillance system in that it captures all deaths, births and migrations within the study area. Hence the status (dead or alive) of the total population at any time point is known and can thus contribute to the numerators and denominators used in calculating mortality rates. Further, demographic surveillance often utilise VA to obtain cause-specific mortality data in the population under surveillance. This is even more crucial in populations where majority of deaths occur outside a health facility and hence analysis based on hospital/health facility data will not give a complete or accurate picture of the mortality profile in the general population. About 70 percent of deaths in the study population occur outside a health facility (APHRC, unpublished data).

## Conclusion

In conclusion, while HIV/AIDS remains a major cause of death among the urban poor as compared to other causes of mortality, there is evidence that HIV mortality has decreased significantly at the population level, and, this decline corresponds with the estimated period of scaling up of the national ART program in Kenya. The slums of Nairobi have limited access to quality health care and exhibit a higher HIV prevalence compared to the national or city-level estimates. Despite their disadvantaged position, slum residents seem to have benefited from the public sector scale-up of ART. We recommend further studies that incorporate ART coverage data in mortality estimates where available. Such information will enhance our understanding of the full impact of ART scale-up in reducing adult mortality among marginalized slum populations.

## Competing interest

The authors declare that they have no competing interests.

## Authors’ contributions

SOO did the literature review, methodology and wrote the first draft of the manuscript. MM developed data analysis plan and methodology, contributed to the interpretation of findings and writing of the paper. GSM participated in coding verbal autopsies, interpretation of findings and writing of the paper. TE did the data analysis and contributed to the interpretation of findings and writing of the paper. AE contributed to interpretation of the findings and writing of the paper. CK participated in coding verbal autopsies, contributed to data analysis plan, interpretation of the findings and writing of the paper. All authors read and approved the final manuscript.

## Pre-publication history

The pre-publication history for this paper can be accessed here:

http://www.biomedcentral.com/1471-2458/13/588/prepub

## Supplementary Material

Additional file 1 All-cause and HIV-related mortality rates by period, gender and age group (after imputation). Click here for file
